# NecroX-5 ameliorates bleomycin-induced pulmonary fibrosis via inhibiting NLRP3-mediated epithelial–mesenchymal transition

**DOI:** 10.1186/s12931-022-02044-3

**Published:** 2022-05-20

**Authors:** Li Min, Zhang Shu-Li, Yuan Feng, Hu Han, Li Shao-Jun, Tong Sheng-Xiong, Tian Jia-Yu, Fang Xiang-Zhi, Feng Dan

**Affiliations:** 1 Department of Pain Management, Wuhan First Hospital, Wuhan, China; 2 Department of Critical Care Medicine, Union Hospital, Tongji Medical College, Huazhong University of Science and Technology, Wuhan, China

**Keywords:** NecroX-5, Lung injury, Fibrosis, EMT, NLRP3

## Abstract

**Background:**

Pulmonary fibrosis is a progressive and usually lethal pulmonary disease. Despite considerable research efforts, no effective therapeutic strategy for pulmonary fibrosis has been developed. NecroX-5 has been reported to possess anti-inflammatory, anti-oxidative and anti-tumor activities. In the present study, we aimed to determine whether NecroX-5 exhibits antifibrotic property in bleomycin (BLM)-induced pulmonary fibrosis.

**Results:**

We found that pre-treatment with NecroX-5 alleviated inflammatory response, reduced oxidative stress, inhibited epithelial–mesenchymal transition (EMT), and ameliorated pulmonary fibrosis in vivo and in vitro. Our data further indicated that NecroX-5 substantially reduced activation of NLRP3 inflammasome and TGF-β1/Smad2/3 signaling in vivo and in vitro. Additionally, NLRP3 overexpression significantly reversed the protective effects of NecroX-5 in lung epithelial cells exposed to BLM.

**Conclusions:**

Overall, our results demonstrate the potent antifibrotic properties of NecroX-5 and its therapeutic potential for pulmonary fibrosis.

**Supplementary Information:**

The online version contains supplementary material available at 10.1186/s12931-022-02044-3.

## Introduction

Idiopathic pulmonary fibrosis (IPF) is a severe chronic lung disease with a median survival rate of only 4 years [1–3]. Despite widespread efforts, only two FDA-approved drugs, namely, pirfenidone and nintedanib, have been shown to slow the rate of pulmonary function deterioration [4]. Lung transplantation is the only therapeutic method to extend the lives of IPF patients. Therefore, development of novel and effective therapeutic drugs is very crucial for IPF.

IPF is characterized by chronic inflammation, fibroblast foci accumulation and excessive deposition of extracellular matrix [5]. Accumulating evidences have suggested that epithelial–mesenchymal transition (EMT) is one of the most important origins of mesenchymal cells that promote the production of extracellular matrix proteins [6]. Epithelial cells lose the epithelial adhesion protein, accompanied by the acquisition of interstitial cell marker [7, 8]. Although the pathomechanism of IPF is still not fully understood, it is believed that the EMT process may eventually lead to the development of pulmonary fibrosis.

Uncontrolled inflammatory responses and excessive oxidative stress are essential for the pathogenesis of pulmonary fibrosis and EMT. As known, inflammation and oxidative stress are mutually connected [9]. Previous research has established that a reduction in oxidative stress and inhibition of the inflammatory response directly prevent the EMT process and pulmonary fibrosis [10]. NecroX-5 is a cell-permeable necrosis inhibitor that exert antioxidant activity by scavenging reactive oxygen species (ROS) [11]. NecroX-5 and other NecroX compounds are known to be cytoprotective against several insults, including hypoxic injury, oxidative stress and inflammation [12, 13]. Meanwhile, our previous study showed the anti-inflammatory effects of NecroX-5 in septic animal models [13]. Notably, the antifibrotic effects of NecroX-5 in hypoxia/reoxygenation-treated rat hearts have been evaluated [14]. However, the efficacy of NecroX-5 in pulmonary fibrosis remains unreported.

In our study, we found that NecroX-5 inhibited inflammation, oxidative stress, EMT and pulmonary fibrosis in bleomycin (BLM) -treated mice. Furthermore, our findings demonstrated that NecroX-5 ameliorated BLM-induced pulmonary fibrosis by inhibiting the TGF-β1/Smad2/3-mediated EMT process, which is dependent on the inhibition of NLRP3 inflammasomes. This is the first article reporting the anti-fibrotic effects of NecroX-5 on BLM-induced pulmonary fibrosis, which discovers a novel role for NecroX-5 in IPF and provides a possible therapeutic target for IPF treatment.

## Material and methods

### Materials

The information for all reagents is described in Additional file 1.

### Pulmonary fibrosis model in mouse

In this study, 8-week-old fed male C57BL/6 J mice weighing 23–30 g were housed in the SPF environment with free access to food and water. The mice were stabilized for three days and then randomly divided into three groups: the control group, the BLM group, and the BLM with NecroX-5 group (10 mg/kg). The surviving mice were sacrificed separately at 1 day, 3 days, 7 days and 14 days after the operation (n = 6 for each time).

A mouse model of pulmonary fibrosis was established by the intratracheal instillation of BLM as previously described [15]. Animals from the control groups received one intratracheal instillation of the same amount of 0.9% saline. According to our previous study [13], mice in the BLM with NecroX-5 group were pre-treated with NecroX-5 (10 mg/kg of body weight) once daily via intragastric administration for 7 days,. Then, BLM was administered intratracheally (50 μl, 3.0 mg/kg). The NecroX-5 treatments were continued until 1 day, 3 days,7 days or 14 days after BLM administration (Additional file 1: Fig.S1A). All procedures involving animal experimentation were performed in accordance with the ethical guidelines of the Tongji Medical College of Huazhong University of Science and Technology.

**Fig. 1:**
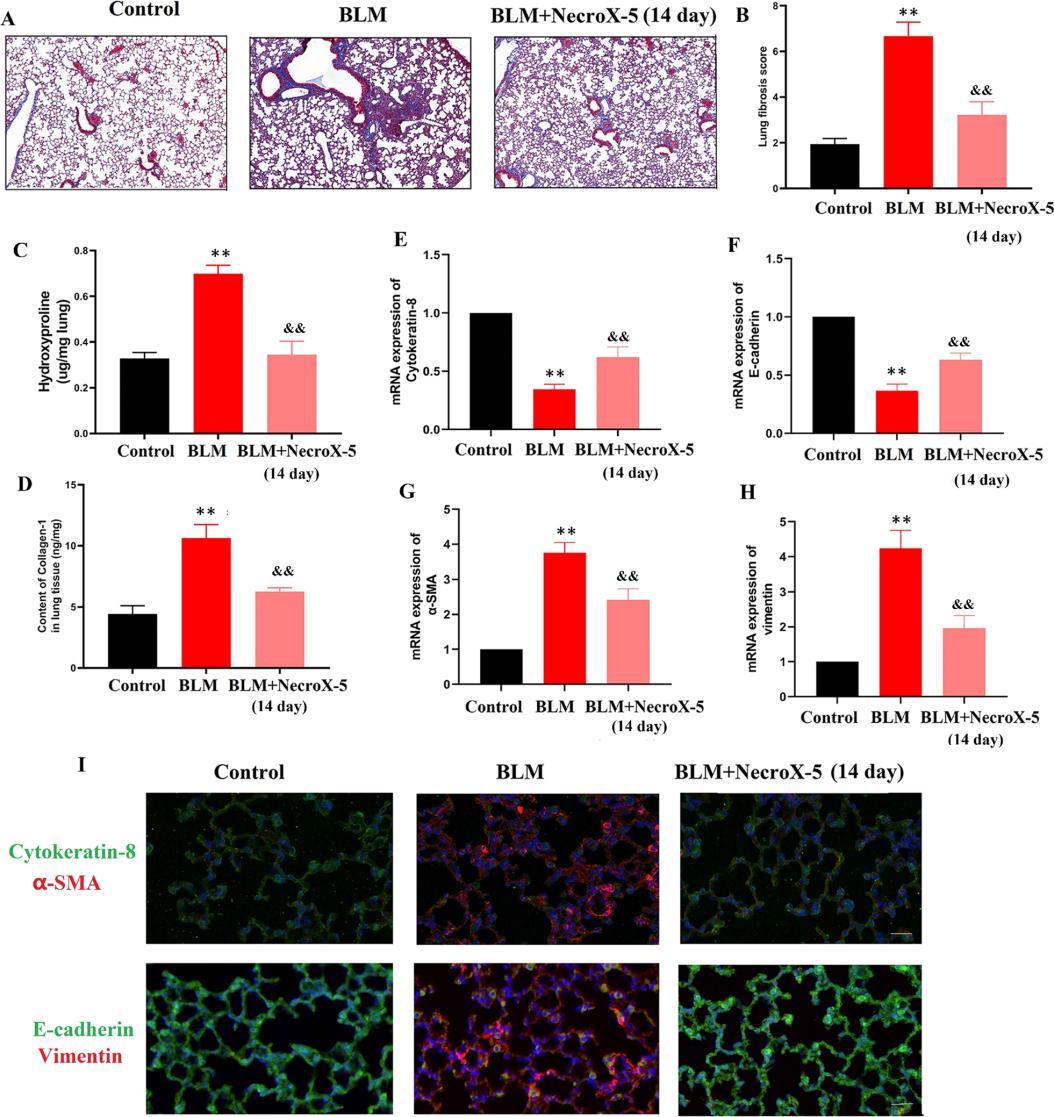
NecroX-5 ameliorated pulmonary fibrosis in BLM-treated mice. **A** Masson’s trichrome staining. **B** Lung fibrosis score. **C** Lung tissue levels of hydroxyproline at Day 14. **D** Lung tissue levels of COL-1. **E** cytokeratin-8 mRNA expression in lung tissue. **F** E-cadherin mRNA expression in lung tissue. **G** α-SMA mRNA expression in lung tissue. **H** vimentin mRNA expression in lung tissue. **I** The expression of epithelial markers (cytokeratin-8, E-cadherin) and mesenchymal markers (α-SMA, vimentin) in lung tissue was confirmed by immunostaining. *P < 0.05, vs. controls; ^&^P < 0.05 vs. BLM

### Cell culture

Mouse epithelial cells (MLE-12) and human lung epithelial cells (BEAS-2B) were purchased from the Cell Bank of the Chinese Academy of Sciences. Both cell lines were cultured in Dulbecco's modified Eagle’s medium/nutrient mixture F-12 (DMEM/F12) containing 10% foetal bovine serum (FBS) at 37 °C with 5% CO_2_. Cells were pre-treated with 0.4 mM NecroX-5 for 2 h, and then stimulated with 50 μM BLM for 12 h, 24 h or 48 h.

An NLRP3-overexpression plasmid was obtained from Shanghai Jikai Company. The empty plasmid was employed as the negative control. Cell transfection was executed using Lipofectamine 2000 reagent in accordance with the manufacturer’s instructions.

### Histological analysis

The left lungs were fixed, embedded, cut, and stained with haematoxylin/eosin. Lung injury scores were assessed according to previous methods [16]. Lung fibrosis were evaluated using Masson’s trichrome staining. The degree severity of lung fibrosis was quantified by Ashcroft scoring system [17].

### Real-time PCR

Total RNA was extracted from lung tissue samples or cells using TRIzol reagent. Next, cDNA was synthesized using a Prime Script™ RT Master Mix Kit. Real-time PCR was performed using a SYBR® Premix Ex Taq™ Kit on an ABI StepOnePlus Real-Time PCR system. The primers used in the study are shown in Additional file 1: Table S1 and Table S2. β-actin was used as an endogenous control.

### Measurement of the levels of hydroxyproline, Collagen 1, TNF-α, IL-1β, TGF-β1 and intracellular ROS

The levels of hydroxyproline, Collagen 1, TNF-α, IL-1β, and TGF-β1 in the lung tissue and cell supernatant were detected by ELISA with commercial kits. ROS production was measured using DCFDA by the manufacturer’s instructions.

### Detection of mitochondrial ROS

Cells were loaded with the fluorogenic probe MitoSOX™ Red (3 μmol/L) for 20 min. After removing MitoSOX™ Red and washing cells with Hanks’ balanced salt solution. Cells were viewed with a fluorescence microscope (Nikon, Tokyo, Japan).

### Measurement of the levels of MPO, MDA and SOD.

The lung tissue MPO, SOD and MDA levels were measured using commercial kits, according to the manufacture instructions. According to previous study [18], mitochondrial matrix was prepared from mitochondria by freezing and defrosting with repeated homogenization in order to burst mitochondria. After centrifugation at 10,000*g* for 10 min, the supernatant was considered as the source of mitochondrial MDA. In other hand, lung tissues were homogenized in three volumes of phosphate buffer 0.1 M with KCl 1.17% (ph7.4) and centrifuged at 2000*g* for 15 min. The resultant supernatant was used to determine levels of cytosolic MDA.

### Immunofluorescence staining

Immunofluorescence was carried out according to a previous study [18]. Briefly, cells were fixed with 4% paraformaldehyde and blocked with 3% BSA for 15 min. For lung tissue, after deparaffinization, sections were blocked with normal goat serum for 40 min. Then, cells or lung tissue sections were incubated overnight with primary antibodies followed by fluorescein-conjugated secondary antibody. After washing, the cells or lung tissue sections were incubated with DAPI for 10 min. Cell or sections were viewed with a fluorescence microscope (Nikon, Tokyo, Japan).

### Western blotting

Firstly, protein was extracted using RIPA lysis buffer (Beyotime, Nanjing, China). Then, extracted protein samples (30 µg/lane) were separated via SDS–PAGE and transferred onto PVDF membranes. Then, membranes were incubated with primary antibody (Additional file 1: Table S3) at 4 °C overnight. followed by an HRP-conjugated secondary antibody. After the membranes were washed in TBST, the bands were detected by ECL (Agilent, China).

### Immunofluorescence staining

Immunofluorescence was performed according to our previous study. Briefly, the cells were fixed with 4% paraformaldehyde for 15 min and blocked with 3% BSA for 15 min. For lung tissue, after deparaffinization, sections were blocked with normal goat serum. Then, the cells or lung tissue sections were incubated overnight with primary antibodies (Additional file 1: Table S3), after which the cells or lung tissue sections were incubated with Alexa Fluor 488-labelled goat anti-rabbit IgG or Alexa Fluor 594-labelled goat anti-rabbit secondary antibodies (1:200) for 60 min in the dark. After washing, the cells or lung tissue sections were incubated with DAPI for 10 min. The sections or cells were viewed with a fluorescence microscope (Nikon, Tokyo, Japan).

### Transmission electron microscopy (TEM)

After treatment, cells were fixed in 4% glutaraldehyde for 24 h at 4 °C. Samples were incubated with 1% osmium tetroxide, after which they were alcohol dehydrated, and araldite embedded. Thin sections (85 nm) were stained with uranyl acetate and lead citrate. Ultrastructural analysis was conducted via TEM (FEI, Hillsboro, Oregon) at 80 kV.

### Statistical analysis

Sample sizes were the estimates based on experience and preliminary data. Except for the evaluation of the lung injury score, investigators were not blinded during data collection or analysis. No data was excluded or missing. The distribution of all data was tested for normality with Shapiro–Wilk tests. Data are expressed as the mean ± SD. One-way analysis of variance (ANOVA) followed by Tukey’s test was performed using GraphPad Prism 5 (GraphPad Software, La Jolla, CA, USA). A two-tailed *P* value less than 0.05 was considered statistically significant.

## Results

### NecroX-5 ameliorated pulmonary damage in BLM-treated mice

In order to investigate the effects of NecroX-5 in pulmonary fibrosis, a mouse pulmonary fibrosis model was constructed using BLM. HE staining showed severe lung damage characterized by structural confusion of the lung tissue and mononuclear infiltration in mice exposed to BLM. After NecroX-5 pre-treatment, the lung damage induced by BLM was significantly ameliorated at 1 day, 3 days, 7 days, and 14 days (Additional file 1: Fig. S1B and 1C).

Increasing data have demonstrated that excess oxidative stress and inflammation take part in the process of BLM-evoked lung fibrosis. To investigate whether NecroX-5 mediated the inhibition of lung inflammation in mice exposed to BLM, we then determined the production of proinflammatory cytokines in lung tissue. The levels of TNF-α and IL-1β in the lung were significantly increased in mice exposed to BLM. Administration of NecroX-5 effectively reduced the levels of TNF-α and IL-1β in lung tissue (Additional file 1: Fig. S1D and E). Additionally, we examined whether NecroX-5 affects neutrophil infiltration. Our results showed that neutrophils in BALF and MPO activity in lung tissue were decreased after pre-treatment with NecroX-5 (Additional file 1: Fig. S1F and G). We then set out to determine whether NecroX-5 affects oxidative stress in mice exposed to BLM. We found that SOD levels in the lungs were decreased after BLM treatment. After pre-treatment with NecroX-5, BLM-induced decreases in SOD were inhibited in mouse lungs at 1 day, 3 days, 7 days, and 14 days (Additional file 1: Fig. S1H). Simultaneously, the level of MDA in cytoplasm and mitochondria was examined as well. After pre-treatment with NecroX-5, BLM-induced increases in MDA were inhibited in cytoplasm and mitochondria at 1 day, 3 days, 7 days, and 14 days (Additional file 1: Fig. S1I and J).

Taken together, these results demonstrate that NecroX-5 ameliorates pulmonary damage in vivo.

### NecroX-5 ameliorated pulmonary fibrosis in BLM-treated mice

The degree of fibrosis was evaluated using Masson’s trichome staining. We found that BLM increased the deposition of fibrillar collagen, which is consistent with the Ashcroft score. NecroX-5 treatment for 14 days significantly attenuated fibrillar collagen deposition and decreased the Ashcroft score in mice exposed to BLM (Fig. 1A and B). Additionally, we examined whether NecroX-5 affects the levels of hydroxyproline and collagen I, biomarkers of fibrosis. Our results showed that BLM significantly upregulated the levels of hydroxyproline and collagen I, which were inhibited by NecroX-5 (Fig. 1C and D). Taken together, these results demonstrate that NecroX-5 ameliorates pulmonary damage and fibrosis induced by BLM.

### NecroX-5 inhibited the EMT process in BLM-treated mice

E-cadherin and cytokeratin-8 are well-known biomarkers of epithelial cells, and vimentin and α-SMA are biomarkers of mesenchymal cells [19]. We found that the mRNA expression levels of E-cadherin and cytokeratin-8 were decreased (Fig. 1E and F), whereas the mRNA expression levels of α-SMA and vimentin were increased in mice exposed to BLM (Fig. 1G and H). These changes were reversed by NecroX-5 at 14 days (Fig. 1D and E). Immunofluorescence staining demonstrated similar changes in the epithelial cell marker and the myofibroblast marker (Fig. 1I). These results indicated that NecroX-5 could suppress the EMT process in BLM-treated mice.

### NecroX-5 inhibited inflammation and oxidative stress in pulmonary epithelial cells exposed BLM

To determine whether NecroX-5 inhibited the production of proinflammatory cytokines in vitro, we evaluated the expression of TNF-α and IL-1β in culture medium by ELISA. We found that pre-treatment with NecroX-5 significantly reduced the level of TNF-α and IL-1β in MLE-12 cells and BEAS-2B cells stimulated with BLM (Additional file 1: Fig. S2A-D). These data indicated that NecroX-5 could eliminate BLM-induced proinflammatory cytokine elevation in vitro.

Next, we explored whether NecroX-5 also affected cellular ROS. As illustrated in Additional file 1: Fig. S2E, BLM treatment resulted in notable increases in intracellular ROS levels. Nevertheless, pre-treatment with NecroX-5 significantly inhibited the abnormality of ROS in MLE-12 cells and BEAS-2B cells stimulated with BLM. Simultaneously, the level of mitochondrial ROS was examined as well. After pre-treatment with NecroX-5, BLM-induced increases in mitochondrial ROS were inhibited (Additional file 1: Fig. S2F). In addition, mitochondrial morphology was detected by TEM. Our results showed that mitochondria in MLE-12 cells and BEAS-2B cells stimulated with BLM underwent fission, which resulted in smaller mitochondria (Additional file 1: Fig. S2G). However, NecroX-5 had no significant impact on the change of mitochondrial morphology. This indicated that NecroX-5 could suppress ROS production, but not inhibit the change of mitochondrial morphology in pulmonary epithelial cells after BLM stimulation.

### NecroX-5 inhibited fibrosis and EMT in pulmonary epithelial cells exposed BLM

**Fig. 2:**
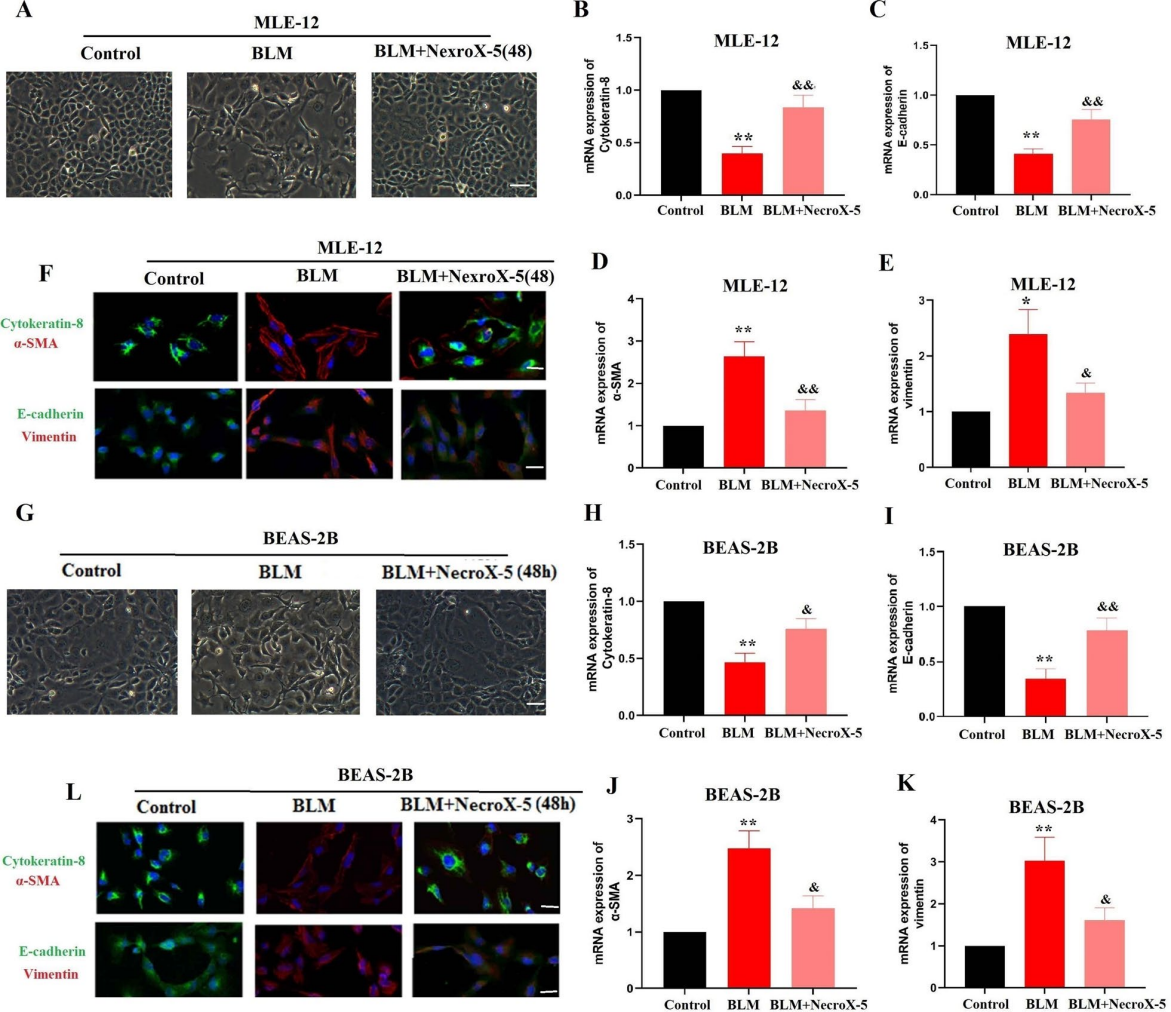
NecroX-5 inhibited the EMT process in pulmonary epithelial cells exposed to BLM.** A** The morphology of MLE-12 cells. **B** Cytokeratin-8 mRNA expression in MLE-12 cells. **C** E-cadherin mRNA expression in MLE-12 cells. **D** α-SMA mRNA expression in MLE-12 cells. **E** Vimentin mRNA expression in MLE-12 cells. **F** The expression of epithelial markers and mesenchymal markers in MLE-12 cells was confirmed by immunostaining.** G** The morphology of BEAS-2B cells. **H** Cytokeratin-8 mRNA expression in BEAS-2B cells. **I** E-cadherin mRNA expression in BEAS-2B cells. **J** α-SMA mRNA expression in BEAS-2B cells. **K** Vimentin mRNA expression in BEAS-2B cells. **L** The expression of epithelial markers and mesenchymal markers in BEAS-2B cells was confirmed by immunostaining. *P < 0.05, vs. controls; ^&^P < 0.05 vs. BLM

In order to explored the role of NecroX-5 in BLM-induced pulmonary fibrosis in vitro, we used BEAS-2B cells and MLE-12 cells to establish a pulmonary fibrosis model in vitro. First, we observed the morphology of the cells. We found that the morphology of BEAS-2B cells and MLE-12 cells changed from round or oval into a long spindle shape with increased intercellular space. However, NecroX-5 reversed the effect of BLM on the morphology of the cells (Fig. 2A and G).

Furthermore, we investigated the effects of NecroX-5 on the EMT process in pulmonary epithelial cells after BLM stimulation. Both BEAS-2B cells and MLE-12 cells stimulated with BLM expressed higher mRNA levels of α-SMA and vimentin as well as lower mRNA levels of E-cadherin and cytokeratin-8 (Fig. 2B–E and Fig. 2H–K), which is consistent with what was observed in vivo. In addition, NecroX-5 could reverse these changes. Immunofluorescence staining in BEAS-2B and MLE-12 cells demonstrated similar changes in the epithelial cell marker and the myofibroblast marker (Fig. 2F and L). Therefore, NecroX-5 reversed the BLM-induced EMT process in pulmonary epithelial cells after BLM stimulation.

### NecroX-5 inhibited NLRP3 activation and the TGF-β1/Smad2/3 pathway in BLM-treated mice

**Fig. 3:**
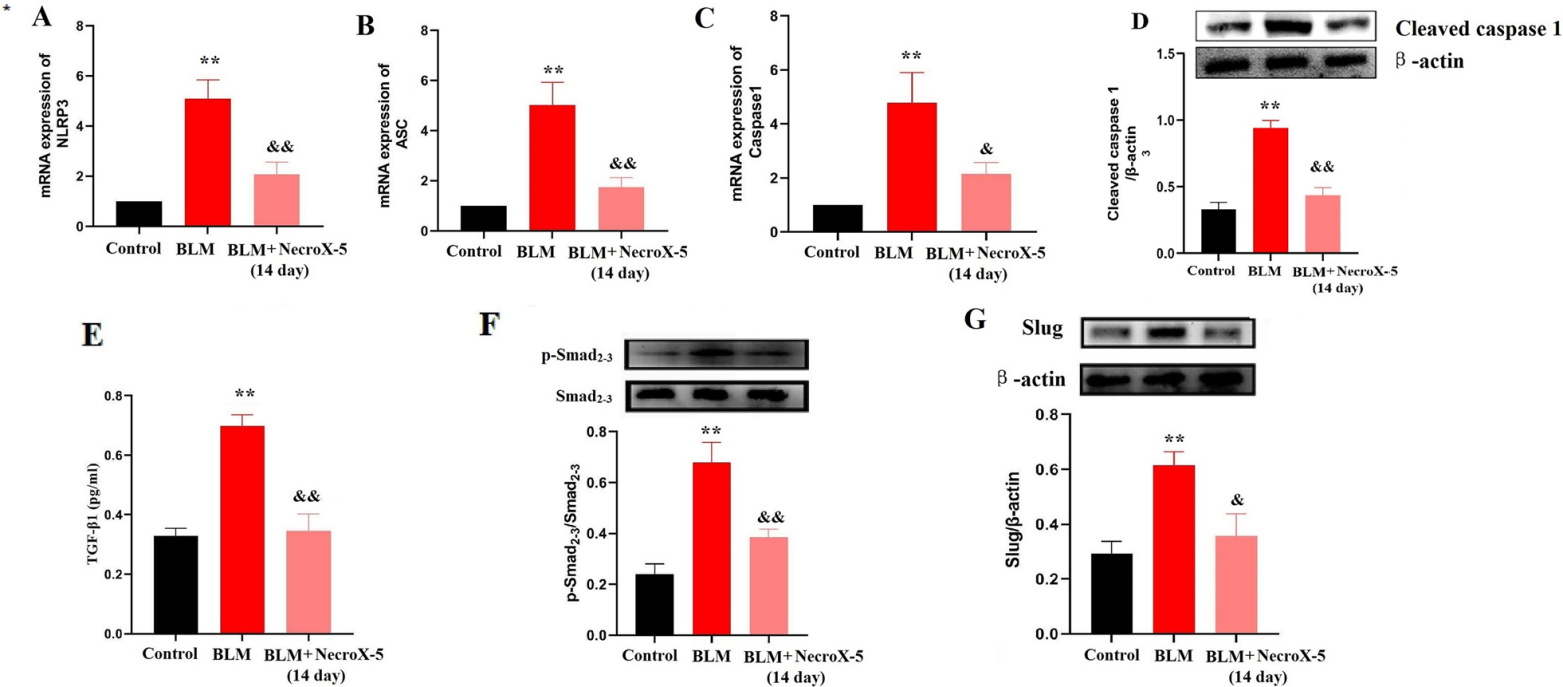
NecroX-5 inhibited NLRP3 activation and the TGF-β1/Smad2/3 pathway in BLM-treated mice. **A** NLRP3 mRNA expression in lung tissue. **B** ASC mRNA expression in lung tissue. **C** Caspase-1 mRNA expression in lung tissue. **D** The expression of cleaved caspase 1 in lung tissue. **E** Lung tissue levels of TGF-β1. **F** Protein expression of phospho (p-)Smad2/3. **G** Protein expression of Slug. *P < 0.05. vs. controls; ^&^P < 0.05 vs. BLM

Previous studies demonstrated that NecroX-5 suppressed the NLRP3 pathway, which contributes to lung fibrosis in mice [13, 20, 21]. Thus, we examined the expression of NLRP3 inflammasome genes in vivo. We found that the mRNA levels of NLRP3, Caspase-1, and ASC were increased in the lung tissues of mice exposed to BLM (Fig. 3A–C), similar to the results obtained in previous study [22]. We then examined the effect of NecroX-5 on the NLRP3 inflammasome. Our results showed that NecroX-5 dramatically inhibited the upregulation of NLRP3, Caspase-1, and ASC in BLM-treated mice (Fig. 3A–C). The expression levels of cleaved caspase 1 were evaluated. We found that the expression of cleaved caspase 1 was increased in the lung tissues of mice exposed to BLM, which was inhibited by NecroX-5 (Fig. 3D).

A large number of studies have demonstrated the critical role of the TGF-β1/Smad2/3 signalling pathway in fibrosis [23, 24]. Next, we determined the level of TGF-β1, p-Smad2/3, total (t-)Smad2/3, and the downstream transcription factor Slug in lung tissue. We found that NecroX-5 reduced TGF-β1 expression in lung tissue of mice exposed to BLM (Fig. 3E). Additionally, NecroX-5 also inhibited Smad2/3 phosphorylation and Slug activation in mice exposed to BLM (Fig. 3F and G).

### NecroX-5 inhibited NLRP3 activation and the TGF-β1/Smad2/3 pathway in pulmonary epithelial cells exposed BLM

**Fig. 4:**
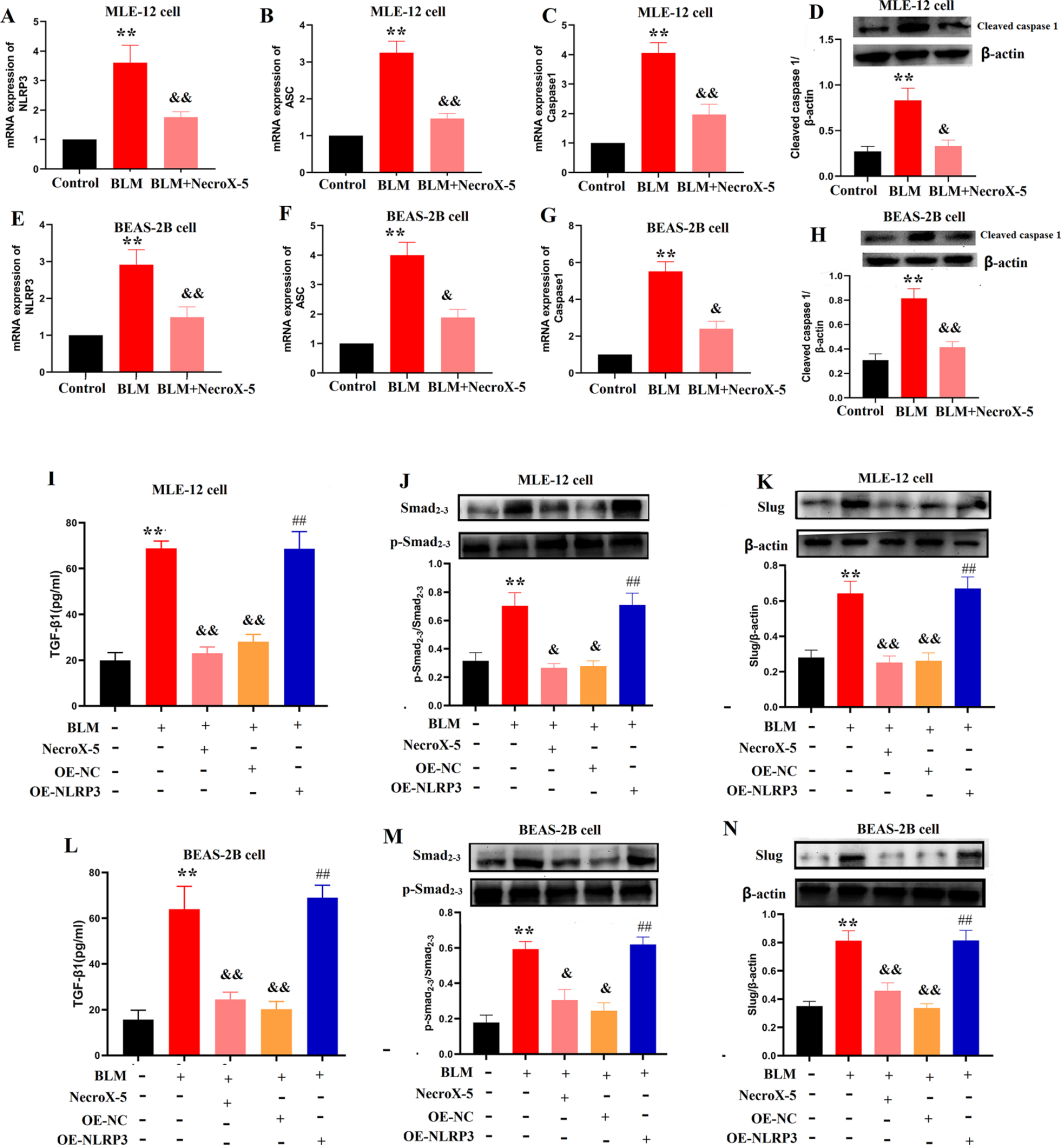
NecroX-5 inhibited the TGF-β1/Smad2/3 pathway depending on NLRP3 activation in pulmonary epithelial cells exposed to BLM. **A** NLRP3 mRNA expression in MLE-12 cells. **B** ASC mRNA expression in MLE-12 cells. **C** Caspase-1 mRNA expression in MLE-12 cells. **D** The expression of cleaved caspase 1 in MLE-12 cells. **E** NLRP3 mRNA expression in BEAS-2B cells. **F** ASC mRNA expression in BEAS-2B cells. **G** Caspase-1 mRNA expression in BEAS-2B cells. **H** The expression of cleaved caspase 1 in BEAS-2B cells. **I** The levels of TGF-β1 in MLE-12 cells. **J** Protein expression of phospho (p-)Smad2/3 in MLE-12 cells. **K** Protein expression of Slug in MLE-12 cells. **L** The levels of TGF-β1 in BEAS-2B cells. **M** Protein expression of phospho (p-)Smad2/3 in BEAS-2B cells. **N** Protein expression of Slug in BEAS-2B cells. *P < 0.05, vs. controls; ^&^P < 0.05 vs. BLM

We then studied whether NecroX-5 affected NLRP3 activation and TGF-β1/Smad2/3 signalling in vitro. We found that NecroX-5 dramatically inhibited the upregulation of NLRP3, Caspase-1, cleaved caspase 1 and ASC in MLE-12 cells and BEAS-2B cells (Fig. 4A-H), consistent with the results observed in vivo. Additionally, NecroX-5 treatment significantly decreased TGF-β1 expression, Smad2/3 phosphorylation and Slug activation in MLE-12 cells and BEAS-2B cells (Fig. 4I–N). These results indicate that NecroX-5 may inhibit lung fibrosis through an NLRP3-dependent pathway in mice.

### The downregulation effect of NecroX-5 on the TGF-β1/Smad2/3 pathway is dependent on NLRP3

Subsequently, to further prove the key role of NLRP3 in the NecroX-5-mediated protective efficacy, we upregulated NLRP3 expression in MLE-12 cells and BEAS-2B cells. The upregulation of NLRP3 was confirmed by western blotting (Additional file 1: Fig. S3). Here, we noted that NLRP3 upregulation reversed the inhibitory effect of NecroX-5 on the TGF-β1/Smad2/3 pathway in MLE-12 cells and BEAS-2B cells (Fig. 4I–N). These data indicated that NecroX-5 inhibited TGF-β1/Smad2/3 pathway by suppressing NLRP3 activation.

### NLRP3 overexpression eliminated the inhibitory effect of NecroX-5 on inflammation, oxidative stress and EMT

**Fig. 5:**
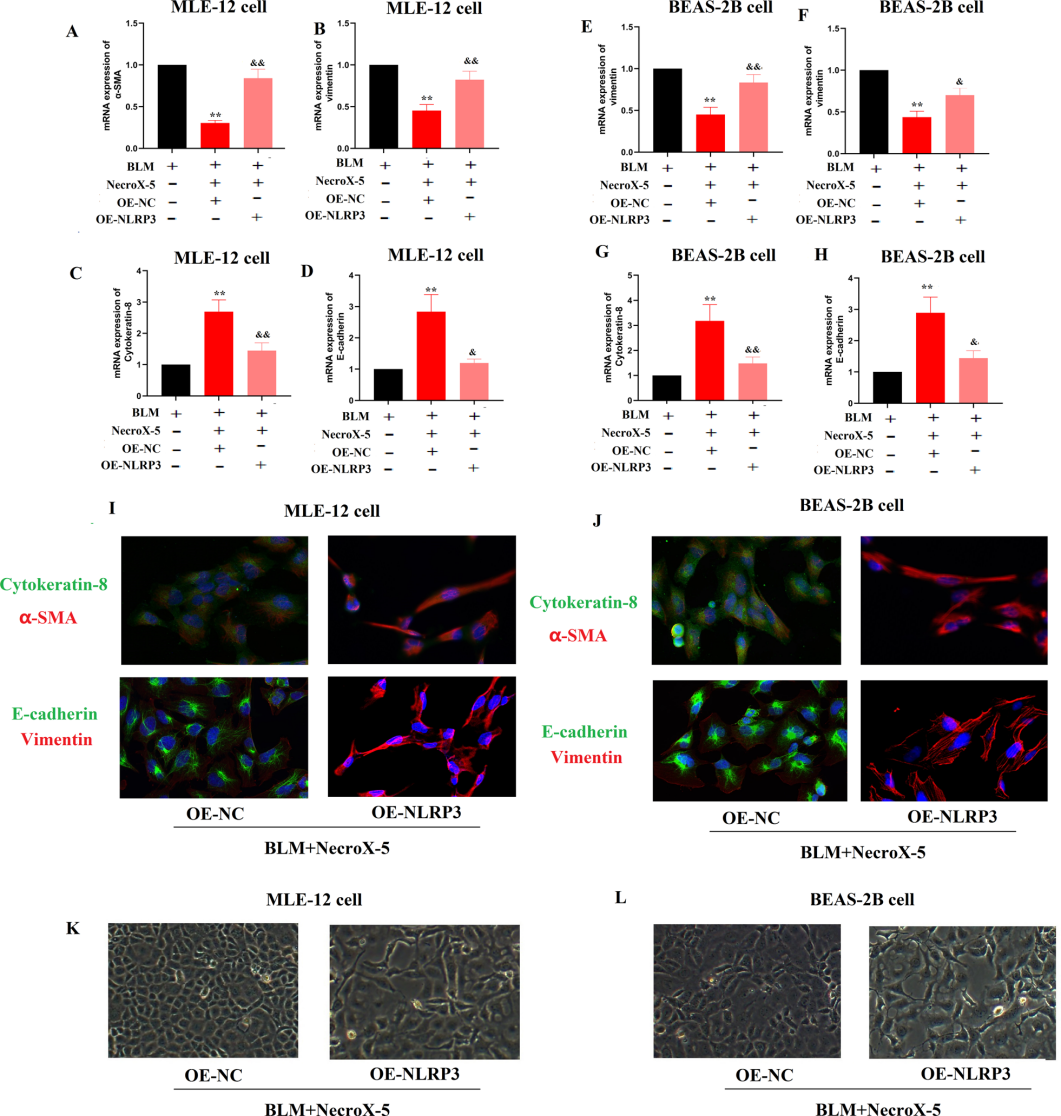
NecroX-5 inhibited EMT process depending on NLRP3 activation in pulmonary epithelial cells exposed to BLM. **A** α-SMA mRNA expression expression in MLE-12 cells. **B** Vimentin mRNA expression in MLE-12 cells. **C** Cytokeratin-8 mRNA expression in MLE-12 cells. **D** E-cadherin mRNA expression in MLE-12 cells. **E** α-SMA mRNA expression expression in BEAS-2B cells.** F** Vimentin mRNA expression in BEAS-2B cells. **G** Cytokeratin-8 mRNA expression in BEAS-2B cells. **H** E-cadherin mRNA expression in BEAS-2B cells. **I** The expression of epithelial and mesenchymal markers in MLE-12 cells was confirmed by immunostaining. **J** The expression of epithelial and mesenchymal markers in BEAS-2B cells was confirmed by immunostaining.** K** The morphology of MLE-12 cells.** L** The morphology of BEAS-2B cells. *P < 0.05, vs. controls; ^&^P < 0.05 vs. BLM

In the end, we explored whether NLRP3 overexpression could diminished anti-inflammatory and anti-fibrotic effects of NecroX-5 in vitro. First, we found that upregulation of NLRP3 effectively increased the IL-1β levels, ROS production in cells treated with BLM and NecroX-5 (S Fig. 4A-F). Second, EMT process inhibited by NecroX-5 were promoted by NLRP3 overexpression. (Fig. 5A–J) Furthermore, upregulation of NLRP3 abrogated the morphological alteration in cells induced by NecroX-5 (Fig. 5K and L). These results supported that NecroX-5 ameliorated BLM-induced pulmonary fibrosis and EMT process by suppression NLRP3 inflammasomes.

## Discussion

Our results showed that NecroX-5 played a protective role against inflammatory responses and oxidative stress induced by BLM in vivo and in vitro. We also provide in vivo and in vitro evidence that NecroX-5 attenuated pulmonary fibrosis and the TGFβ1/Smad2/3-mediated EMT process. Meanwhile, we demonstrated that, in vitro, increasing NLRP3 levels reversed the anti-fibrosis anti-EMT efficacy of NecroX-5. Our results demonstrated for the first time that NecroX-5 could be considered a potential agent for the treatment of pulmonary fibrosis in the future.

Although the pathogenesis of pulmonary fibrosis and EMT process is not definitively identified, but oxidative stress is definitively involved in pathogenesis of IPF [25]. Oxidative stress may be broadly defined as an imbalance between oxidant production and the antioxidant capacity of the cell. This leads to the production of reactive oxygen species (ROS), including hydrogen peroxide (H_2_O_2_), superoxide radical (O_2_ˉ), hydroxyl radical (OH), hypochlorous acid (HOCl), and peroxynitrite (ONOO) [26]. Mitochondria are source of the generation of ROS, and are also the important sites initiating the inflammatory response. Previous studies showed that increased ROS is also a classical mechanism that induces NLRP3 inflammasome activation playing important role in pulmonary fibrosis and EMT [27]. This suggested that NLRP3 inflammasome senses mitochondrial ROS and may explain the frequent association of mitochondrial ROS with pulmonary fibrosis and EMT process.

Pulmonary fibrosis gradually develops during repeated cycles of inflammation and oxidative stress-induced damage and repair. Therefore, reducing the inflammatory response and oxidative stress are the most effective methods for combating pulmonary fibrosis [28]. As a potential agent for anti-inflammation and antioxidative stress, NecroX-5 has been reported to have therapeutic effects in various lung injuries [13]. Further studies revealed the preventive effect of NecroX-5 on the fibrosis responses of hearts in a model of hypoxia/reoxygenation [14].

Our study demonstrated that pre-treatment with NecroX-5 significantly ameliorated lung damage induced by BLM, which was accompanied by the inhibition of oxidative status and inflammation. The results of our study are consistent with previous studies [13]. Epithelial repair after inflammation and oxidative stress is the root of the fibrosis process, characterized by a large deposition of collagens [29, 30]. Here, we revealed that NecroX-5 treatment for 14 days significantly attenuated fibrillar collagen deposition in vivo. These results suggest that NecroX-5 alleviates lung fibrosis by reducing inflammation and oxidative stress.

EMT process is a crucial initiator and contributor to fibrosis [31]. It is characterized by marked changes in cell morphology, loss of epithelial markers such as E-cadherin, and increases in mesenchymal markers such as α-SMA [19]. Our results showed that EMT contributes to the pathogenesis of pulmonary fibrosis, which is in agreement with the results of previous studies [32]. Interestingly, we found that NecroX-5 inhibited BLM-induced EMT in vivo and in vitro, suggesting that NecroX-5 ameliorates pulmonary fibrosis by inhibiting EMT.

The inflammasomes are cytosolic multiprotein complex that play a vital role in innate immunity [33, 34]. The NLRP3 inflammasome composed of NLRP3, ASC and caspase-1, is currently the most extensively studied inflammasome. As a core protein, activated NLRP3 recruits the downstream proteins ASC and procaspase-1 to cause maturation and secretion of the highly proinflammatory cytokines IL-1β and IL-18 [35]. NLRP3 inflammasome activation is also observed in pulmonary fibrosis models induced by asbestos, silica and bleomycin, and pulmonary fibrosis is alleviated by the inhibition of NLRP3 inflammasome activation [36–38]. Our results are in agreement with the results of previous studies, showing that NecroX-5 significantly inhibited NLRP3 inflammasome activation in vivo and in vitro.

In addition, TGF-β1 is considered a crucial mediator in tissue fibrosis and causes tissue scarring largely by activating downstream Smad signalling [39]. Previous study has confirmed that Smad2 and Smad3 are the primary mediators of TGF-β1 signalling [40]. Since phosphorylation of Smad2/3 is the key step of the Smad signalling pathway [41], we chose phosphorylated Smad2/3 as a biomarker to measure the activation of the TGF-β1 signalling pathway. In the present study, we found that NecroX-5 significantly inhibited the TGF-β1/Smad2/3 signalling pathway in response to BLM. It was demonstrated previously that TGF-β1/Smad2/3 signalling activates the expression of Slug, a key transcription factor required for EMT [42]. Consistently, our results revealed that NecroX-5 dramatically inhibited Slug expression.

Several studies have reported the critical role of NLRP3 inflammasome in mediating EMT and TGF-β1 signalling in renal and liver fibrosis [22, 43]. Thus, we inferred that NecroX-5 alleviated TGF-β1/Smad2/3 signalling medicated EMT process and pulmonary fibrosis associating with BLM by suppressing NLRP3 inflammasome activation. In order to investigate the key role of NLRP3 inflammasome in antifibrosis efficacy of NecroX-5, we upregulated NLRP3 expression in cell. We found that NLRP3 overexpression abrogated the suppression of TGF-β1/Smad2/3 signalling and EMT by NecroX-5. This suggests that NecroX-5 ameliorated BLM-induced pulmonary fibrosis by inhibiting the TGF-β1/Smad2/3- mediated EMT process, which is dependent on the inhibition of NLRP3 inflammasomes.

In conclusion, for the first time to our knowledge, our study demonstrates that NecroX-5 alleviates BLM-induced lung fibrosis, which sheds a light on a potential drug for patients with IPF.

## Supplementary Information

**Additional file 1: Fig S1.** NecroX-5 ameliorated pulmonary damage in BLM-treated mice. (A) Design of animal experiments. (B). HE staining. (C). Lung injury score. (D). The levels of TNF-α in BALF. (E). The levels of IL-1β in BALF. (F) neutrophil number in BALF. (G) Lung tissue levels of MPO. (H) Lung tissue levels of SOD. (I) Mitochondrial MDA levels in lung tissue. (J) Cytosolic MDA levels in lung tissue. *P < 0.05vs. control; ^&^P < 0.05 vs. BLM. **Fig S2. **NecroX-5 inhibited inflammation and oxidative stress in pulmonary epithelial cells exposed BLM. (A) The levels of TNF-α in MLE-12 cells. (B) The levels of IL-1β in MLE-12 cells. (C) The levels of TNF-α in BEAS-2B cells. (D) The levels of IL-1β in BEAS-2B cells. (E) The levels of intracellular ROS. Bar=10um. (F) The levels of mitochondrial ROS. Bar=10um. (G)mitochondrial morphology by TEM. Bar=1um. *P < 0.05 vs. control; ^&^P < 0.05 vs. BLM. **Fig S3. **The upregulation of NLRP3 was confirmed by western blotting. (A) The expression of NLRP3 in MLE-12 cells. (B) The expression of NLRP3 in MLE-12 cells. **Fig S4.** NLRP3 overexpression eliminated the inhibitory effect of NecroX-5 on inflammation and oxidative stress. (A) The levels of TNF-α in MLE-12 cells. (B) The levels of IL-1β in MLE-12 cells. (C) The levels of TNF-α in BEAS-2B cells. (D) The levels of IL-1β in BEAS-2B cells. (E) The levels of intracellular ROS. Bar=10um. *P < 0.05 vs. OE-NC. **Table S1.** Human primers used and RT-PCR conditions. **Table S2.** Mouse primers used and RT-PCR conditions. **Table S3.** Antibodies used for Western blot, and immunofluorescence.

## Data Availability

The datasets used and/or analyzed during the current study available from the corresponding author on reasonable request.
