# Supplementary material for: NecroX-5 ameliorates bleomycin-induced pulmonary fibrosis via inhibiting NLRP3-mediated epithelial–mesenchymal transition
Source: Respir Res. 2022 May 20;23:128. doi: 10.1186/s12931-022-02044-3 (PMC9121617; doi:10.1186/s12931-022-02044-3)
Supplement: Supplementary file 1 — Additional file 1: Fig S1. NecroX-5 ameliorated pulmonary damage in BLM-treated mice. (A) Design of animal experiments. (B). HE staining. (C). Lung injury score. (D). The levels of TNF-α in BALF. (E). The levels of IL-1β in BALF. (F) neutrophil number in BALF. (G) Lung tissue levels of MPO. (H) Lung tissue levels of SOD. (I) Mitochondrial MDA levels in lung tissue. (J) Cytosolic MDA levels in lung tissue. *P < 0.05vs. control; &P < 0.05 vs. BLM. Fig S2. NecroX-5 inhibited inflammation and oxidative stress in pulmonary epithelial cells exposed BLM. (A) The levels of TNF-α in MLE-12 cells. (B) The levels of IL-1β in MLE-12 cells. (C) The levels of TNF-α in BEAS-2B cells. (D) The levels of IL-1β in BEAS-2B cells. (E) The levels of intracellular ROS. Bar=10um. (F) The levels of mitochondrial ROS. Bar=10um. (G)mitochondrial morphology by TEM. Bar=1um. *P < 0.05 vs. control; &P < 0.05 vs. BLM. Fig S3. The upregulation of NLRP3 was confirmed by western blotting. (A) The expression of NLRP3 in MLE-12 cells. (B) The expression of NLRP3 in MLE-12 cells. Fig S4. NLRP3 overexpression eliminated the inhibitory effect of NecroX-5 on inflammation and oxidative stress. (A) The levels of TNF-α in MLE-12 cells. (B) The levels of IL-1β in MLE-12 cells. (C) The levels of TNF-α in BEAS-2B cells. (D) The levels of IL-1β in BEAS-2B cells. (E) The levels of intracellular ROS. Bar=10um. *P < 0.05 vs. OE-NC. Table S1. Human primers used and RT-PCR conditions. Table S2. Mouse primers used and RT-PCR conditions. Table S3. Antibodies used for Western blot, and immunofluorescence. [file 12931_2022_2044_MOESM1_ESM.doc]

**Supplement**

**NecroX-5 ameliorates bleomycin-induced pulmonary fibrosis via inhibiting NLRP3-mediated epithelial–mesenchymal transition**

***Li Min^1^, Zhang Shu-Li^1^, Yuan Feng^1^, Hu Han^1^, Li Shao-Jun，Tong Sheng-Xiong^1^, Tian Jia-Yu^1^, Fang Xiang-Zhi^2^, Feng Dan^1^.***

1. Department of Pain Management, Wuhan First Hospital.

2. Department of Critical Care Medicine, Union Hospital, Tongji Medical College, Huazhong University of Science and Technology, Wuhan, China.

Li Min and Zhang Shu-Li contributed equally to this work.

Corresponding authors: Department of Pain Management, Wuhan First Hospital (Feng Dan). E-mail addresses: fengdan_pain@126.com.

***1. Materials***

Mice were purchased from Beijing Hua Fu Kang Bioscience Co., Ltd. NecroX-5 and BLM were purchased from Med Chem Express Co., Ltd. TNF-α, IL-1β.TGF-β1 and COL-1 ELISA kit were purchased from Wuhan Hualianke Biotechnology Co., Ltd. The MPO, SOD and MDA determination kit were purchased from Jiancheng Bioengineering Institute (Nanjing, China). TRIzol reagent, qRT SuperMix and AceQ SYBR-Green Master Mix were purchased from Vazyme (JiangSu, China). Anti-Cytokeratin-8 antibody and anti-vimentin antibody were purchased from Abcam (Wu Han，USA). Anti-Slug antibody，anti-α-SMA antibody，anti-E-cadherin antibody and anti-Smad2-3 antibody were purchased from Abclonal (Wu Han，China). Anti-p-Smad2-3 antibody was purchased from Proteintech (Wuhan, China). Anti-β-actin antibody were purchased from HuaBio (HangZhou, China). DCFDA was purchased from Shanghai Biyuntian Biological Co., Ltd.


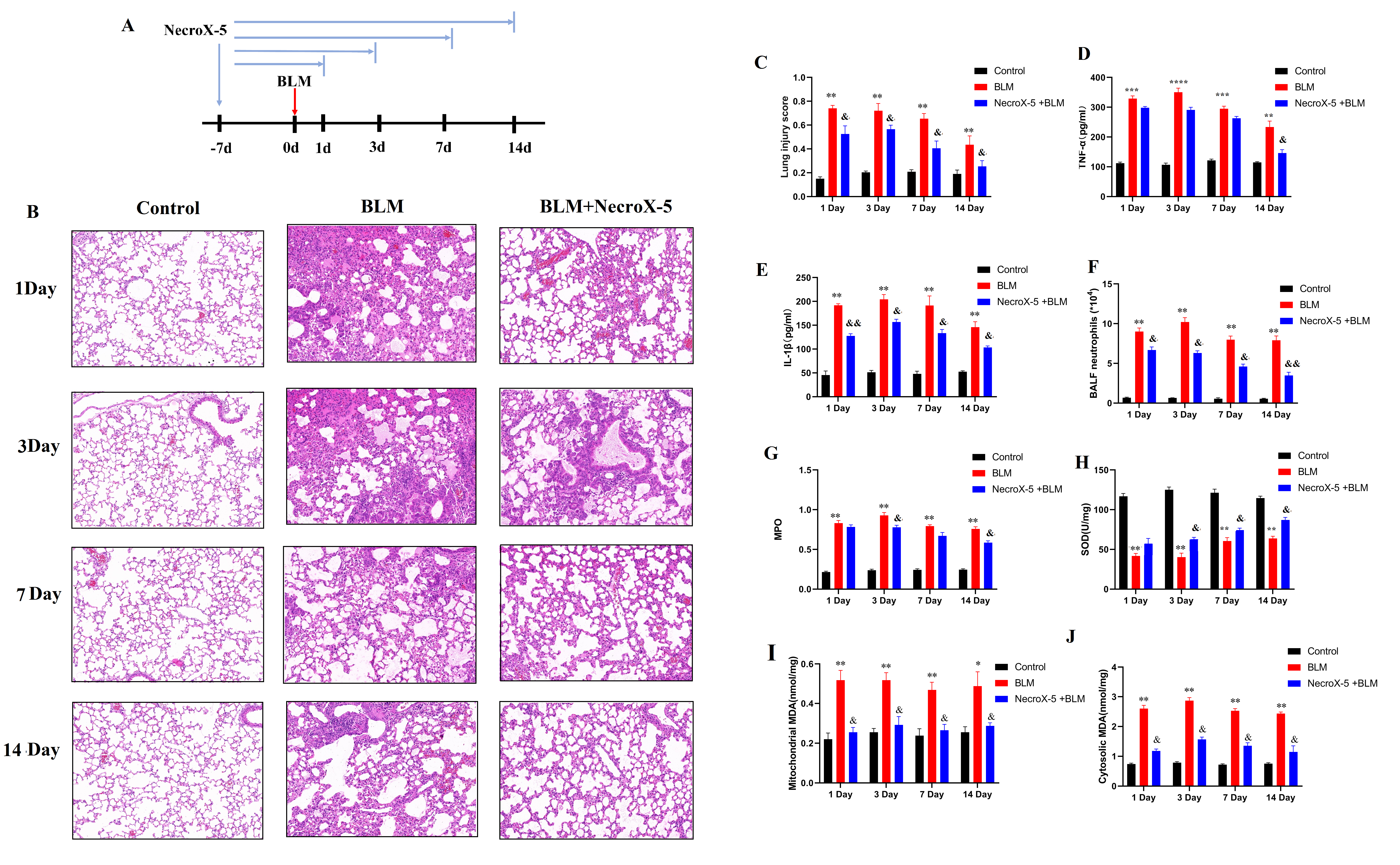
**SFig 1. NecroX-5 ameliorated pulmonary damage in BLM-treated mice.**

1. Design of animal experiments. (B). HE staining. (C). Lung injury score. (D). The levels of TNF-α in BALF. (E). The levels of IL-1β in BALF. (F) neutrophil number in BALF. (G) Lung tissue levels of MPO. (H) Lung tissue levels of SOD. (I) Mitochondrial MDA levels in lung tissue. (J) Cytosolic MDA levels in lung tissue. *P < 0.05vs. control； ^&^P < 0.05 vs. BLM.

**
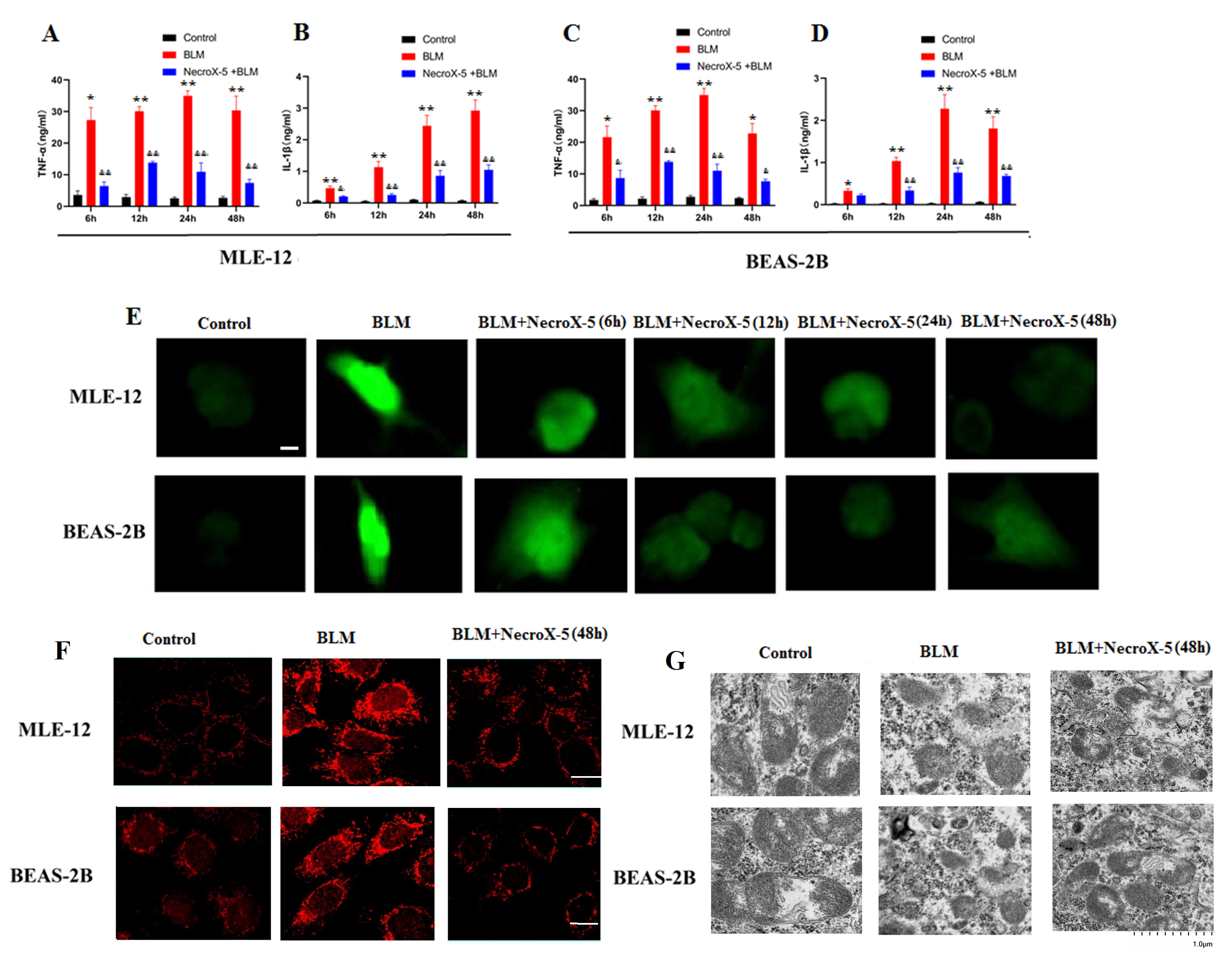
**

**SFig 2. NecroX-5 inhibited inflammation and oxidative stress in pulmonary epithelial cells exposed BLM.**

(A) The levels of TNF-α in MLE-12 cells. (B) The levels of IL-1β in MLE-12 cells. (C) The levels of TNF-α in BEAS-2B cells. (D) The levels of IL-1β in BEAS-2B cells. (E) The levels of intracellular ROS. Bar=10um. (F) The levels of mitochondrial ROS. Bar=10um. (G)mitochondrial morphology by TEM. Bar=1um.

*P < 0.05 vs. control；^&^P < 0.05 vs. BLM.

**
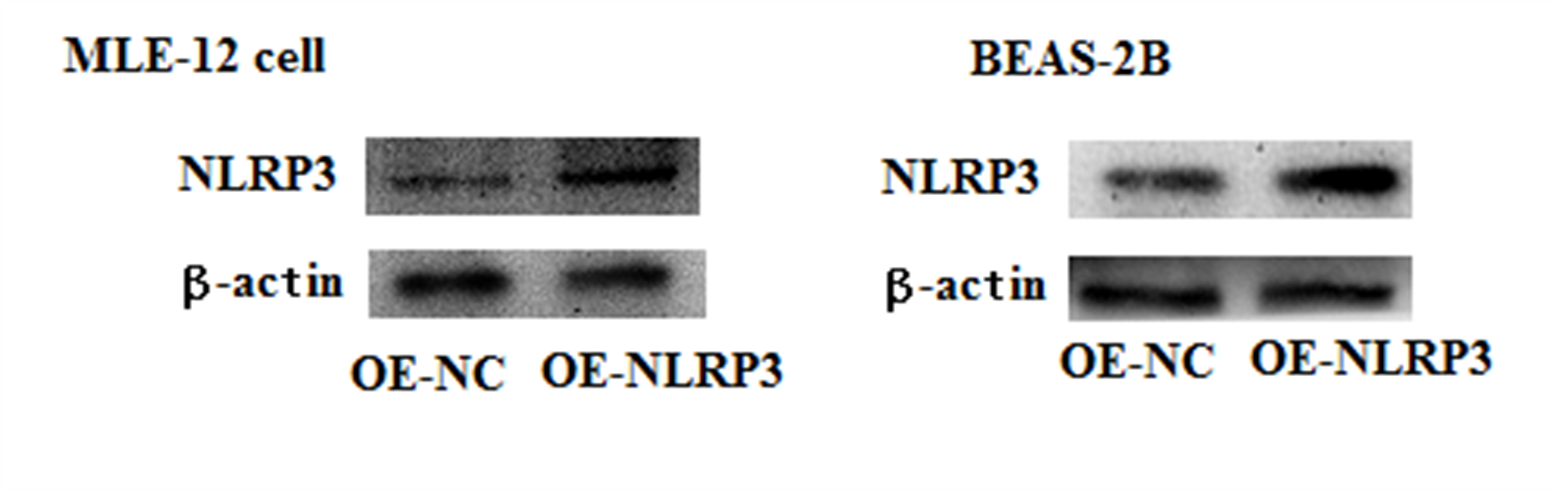
**

**SFig3. The** **upregulation of NLRP3 was confirmed by western blotting.**

(A) The expression of NLRP3 in MLE-12 cells. (B) The expression of NLRP3 in MLE-12 cells.

**
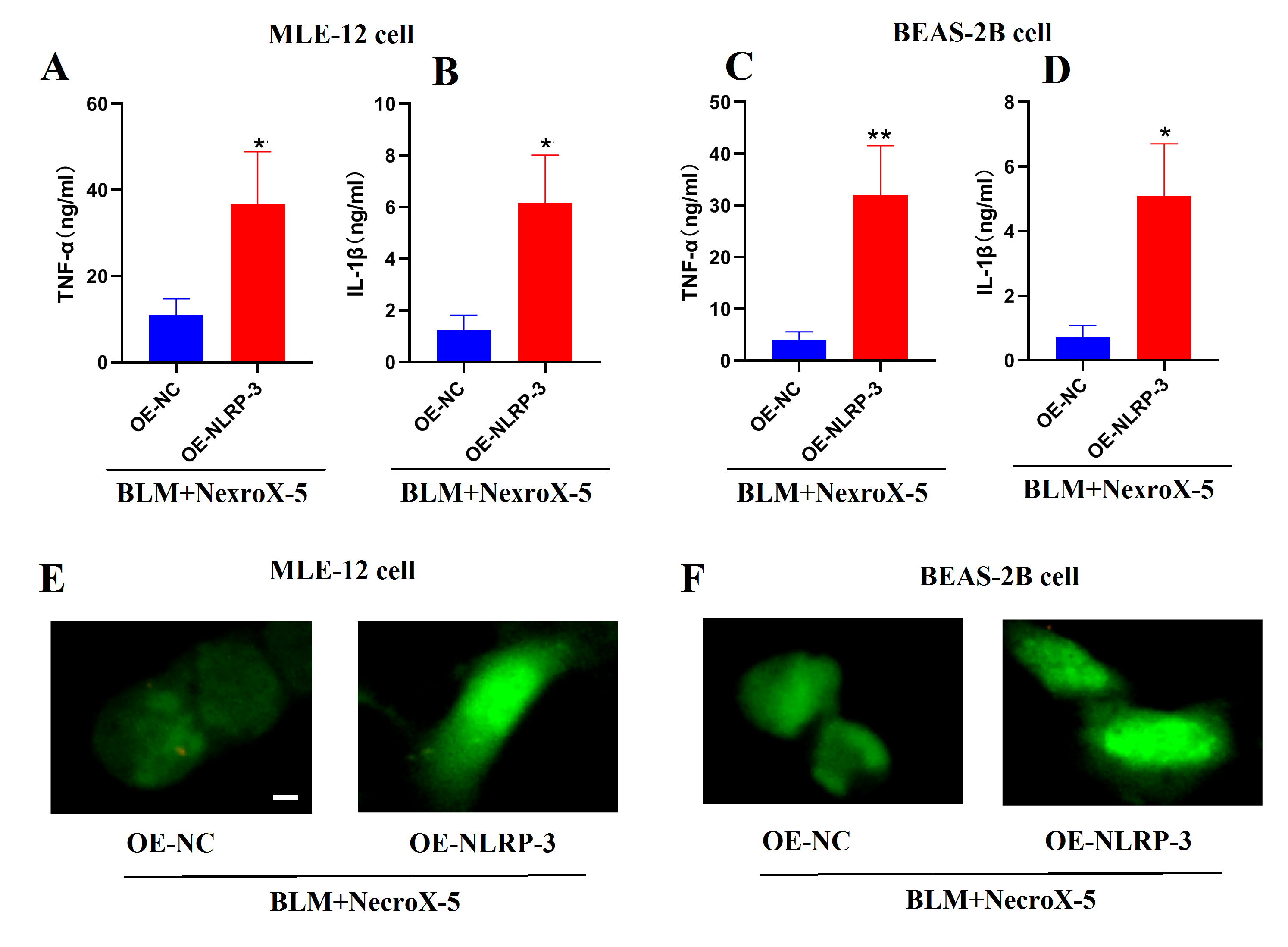
**

**SFig.NLRP3 overexpression eliminated the inhibitory effect of NecroX-5** **on inflammation and oxidative stress.**

(A) The levels of TNF-α in MLE-12 cells. (B) The levels of IL-1β in MLE-12 cells. (C) The levels of TNF-α in BEAS-2B cells. (D) The levels of IL-1β in BEAS-2B cells. (E) The levels of intracellular ROS. Bar=10um. *P < 0.05 vs. OE-NC.

**Table 1. Human primers used and RT-PCR conditions**

| Gene | Sequence |
| --- | --- |
| Cyokeratin-8 | Forward: GAGGCATCACCGCAGTTAC  Reverse: TTGCTTCGAGCCGTCTTCT |
| α-SMA | Forward: CCGACCGAATGCAGAAGGA  Reverse: ACAGAGTATTTGCGCTCCGAA |
| E-cadherin | Forward: GCCAAAGACAGAGCGGAACTAT  Reverse: ATGTGTTCAGCTCAGCCAGC |
| Vimentin | Forward: TGCCGTTGAAGCTGCTAACTAC  Reverse: TAGGTGGCAATCTCAATGTC |
| NLRP3 | Forward: GGACTATTTCCCCAAGATTG  Reverse: ACTCCACCCGATGACAGTT |
| ASC | Forward:CTCACCGCTAACGTGCTGC  Reverse: CTTGGCTGCCGACTGAGGA |
| Caspase-1 | Forward:GGGCTCTGTTTTTATTGGAA  Reverse: ATCTGGCTGCTCAAATGAA |
| β-actin | Forward: AATTGCTTCCACAATCCGAAC  Reverse: TGCTGTCACCTTCACCGTTC |

**Table 2.** Mouse primers used and RT-PCR conditions

| Gene | Sequence |
| --- | --- |
| Cytokeratin-8 | Forward:ATGCAGGGCCTGGTGGAGGA  Reverse: GCCTCAGCTCGGCTGCGATT |
| E-Cadherin | Forward:CCAGTTTCCTCGTCCGCGCC  Reverse:GCTCCTTGGCCGGTGATGCT |
| Vimentin | Forward:ACCTCACTGCTGCCCTGCGT  Reverse:CTCATCCTGCAGGCGGCCAA |
| α-SMA | Forward:AGCGTGGCTATTCCTTCGTGACT  Reverse: CATGGTGCCTGGGTGCGAGGG |
| NLRP3 | Forward:CCACAGTGTAACTTGCAGAAGC  Reverse: GGTGTGTGAAGTTCTGGTTGG |
| ASC | Forward:AAAGAAGAGTCTGGAGCTGTGG  Reverse: GCAATGAGTGCTTGCCTGT |
| Caspase-1 | Forward:CACAGCTCTGGAGATGGTGA  Reverse: GGTCCCACATATTCCCTCCT |
| β-Actin | Forward: TGTTACCAACTGGGACGACA  Reverse: TCTCAGCTGTGGTGGTGAAG |

**Table 3: Antibodies used for Western blot, and immunofluorescence**

| **Target** | **Assay** | **Company** |
| --- | --- | --- |
| E-cadherin | WB (1:1000)  IF(1:200) | Abclonal |
| Cytokeratin-8 | WB (1:1000) | Abcam |
| Cytokeratin-8 | IF(1:200) | Abcam |
| α-SMA | WB (1:1000)  IF(1:200) | Abclonal |
| Vimentin | WB (1:1000) | Abclonal |
| Vimentin | IF(1:200) | Abcam |
| β-actin | WB (1:1000) | Abclonal |

WB: Western Blot, and IF: Immunofluorescence
